# Cancer Prevention at Work (CPW) project: Rationale, framework and research protocol

**DOI:** 10.1371/journal.pone.0335752

**Published:** 2025-11-03

**Authors:** Magdalena Kostrzewa, Monireh Sadat Seyyedsalehi, Alessandro Godono, Giulia Collatuzzo, Giulia Fiorini, Serena Moscato, Ariele Santello, Catharina Roth, Michel Wensing, Giulia Massimino, Maria Teresa Cella, Valentina Biagioli, Mario Magaña, Felipe Augusto Pinto-Vidal, Zuzana Klöslová, Jana Oravec Bérešová, Daniele Bruno, Stefano Giordani, Miriam Kočtúchová Blažinová, Robert Vilček, Marina Ruxandra Otelea, Florina Georgeta Popescu, Iulia Crull, Renata Gili, Egle Gorra, Guillermo Fernandez-Tardon, Marta Maria Rodriguez-Suarez, Violeta Calota, Søren Askegaard, Ángel Honrado, Adonina Tardon, Eleonóra Fabiánová, Daniel Vencovsky, Anna Schneider-Kamp, Dana Mates, Sabato Mellone, Paolo Boffetta

**Affiliations:** 1 Department of Medical and Surgical Sciences, University of Bologna, Bologna, Italy; 2 Department of Public Health and Pediatrics, University of Turin, Turin, Italy; 3 Department of Electrical, Electronic and Information Engineering “Guglielmo Marconi” (DEI), University of Bologna, Bologna, Italy; 4 Health Sciences and Technologies – Interdepartmental Center for Industrial Research (CIRI-SDV), University of Bologna, Bologna, Italy; 5 Heidelberg University, Medical Faculty, Heidelberg, Germany; 6 Department of Prevention and Public Health, Regione Emilia-Romagna, Emilia-Romagna, Italy; 7 WeDo | Project Intelligence Made Easy, S.L., Barcelona, Spain; 8 RPA Europe Prague s.r.o., Prague, Czech Republic; 9 Department of Occupational Health, Regional Authority of Public Health, Banská Bystrica, Slovakia; 10 Onconauti Association, Metropolitan Oncology Network, Bologna, Italy; 11 Occupational Health Service, Železiarne Podbrezová a.s., Podbrezová, Slovakia; 12 Department of Clinical Occupational Medicine and Clinical Toxicology, F. D. Roosevelt Hospital Banská Bystrica, Banská Bystrica, Slovakia; 13 Clinical Department 5, University of Medicine and Pharmacy Carol Davila, Bucharest, Romania; 14 Department of Occupational Health, “Victor Babeş” University of Medicine and Pharmacy, Timișoara, Romania; 15 Romanian Society of Occupational Medicine, Bucharest, Romania; 16 Local Health Unit in Turin, Department of Prevention, Turin, Italy; 17 Intesa San Paolo SpA – Health Surveillance And Psycho-social Risks, Milan, Italy; 18 Health Research Institute of Asturias (ISPA) and University of Oviedo, Oviedo, Asturias, Spain; 19 National Institute of Public Health, Bucharest, Romania; 20 Department of Business and Management, University of Southern Denmark, Odense, Denmark; 21 Stony Brook Cancer Center, Stony Brook University, Stony Brook, New York, United States of America; 22 Department of Family, Population and Preventive Medicine, Renaissance School of Medicine, Stony Brook University, Stony Brook, New York, United States of America; Istituto Nazionale Tumori IRCCS Fondazione Pascale, ITALY

## Abstract

Chronic infections such as *Helicobacter pylori* (Hp), *Hepatitis C virus* (HCV), and *Human Papillomavirus* (HPV) significantly contribute to the global cancer burden, necessitating targeted and cost-effective prevention strategies. The Cancer Prevention at Work (CPW) project pioneers an innovative approach by integrating primary prevention interventions into occupational health surveillance (OHS) programs, thus leveraging existing workplace infrastructure for a large-scale impact. CPW aims to screen and treat Hp and HCV infections, and promote HPV vaccination, targeting not only workers but also their household members. CPW aims to support accessibility, facilitate earlier detection, and strengthen cancer prevention at a population level. The project employs a micro-elimination strategy for HCV, supports data-driven risk assessment for Hp and HPV, and promotes evidence-based communication to reduce HPV vaccine hesitancy. A comprehensive data management framework ensures harmonized data collection, integration, and cost-effectiveness analysis, aiming at providing robust evidence for policy recommendations. Through pilot studies conducted across four European countries, CPW assesses the feasibility, cost-effectiveness, and economic sustainability, with the potential to inform future occupational cancer prevention initiatives across Europe. This work **s**ummarizes the CPW project’s research framework designed to integrate infection-related cancer prevention into workplace health programs across Europe. It details the project’s goals, methods, and discusses the impact on occupational cancer prevention.

## 1. Introduction

Infection-related cancers account for nearly 13% of the global cancer burden, with chronic infections such as *Helicobacter pylori* (Hp), Hepatitis C virus (HCV), and Human Papillomavirus (HPV) linked to gastric, liver, anogenital and oropharyngeal cancers [1]. These three pathogens alone are responsible for approximately 75% of all infection-attributable cancers [1]. Despite the availability of effective screening, treatment, and vaccination strategies, prevention efforts remain suboptimal due to structural barriers, vaccine hesitancy, and limited public health reach [2,3]. Occupational health surveillance (OHS), particularly in Europe, offers a unique but underutilized platform for expanding cancer prevention. These programs are mandatory in many sectors, occur regularly, and benefit from established trust between workers and occupational health professionals [4,5]. Compared to general population interventions, workplace-based screening offers unique advantages—such as structured monitoring and higher adherence—due to the regularity of occupational health services, which are commonly integrated into workplace systems across many European countries, making prevention more feasible, scalable, and sustainable [5–7]. Integrating infection-related cancer interventions into this infrastructure offers a scalable and cost-effective opportunity to reach working populations and their families.

The Cancer Prevention at Work (CPW) Project addresses this gap by embedding evidence-based interventions within workplace programs [8]. The project integrates screening and treatment for Hp and HCV, along with education and promotion of vaccination for HPV, into routine OHS. This approach aims to advance broader cancer prevention, including infections not directly linked to occupational exposures, by leveraging existing occupational health infrastructure. A key innovation of CPW is its ability to extend prevention efforts beyond workers to reach their family members.

CPW adopts several innovative strategies, including a micro-elimination model for HCV at workplaces, ensuring patient linkage to care and follow-up, stakeholder involvement, identifying and overcoming barriers [9,10]. In addition, the application of the Total Worker Health (TWH) framework [11], which integrates workplace health promotion interventions beyond traditional occupational risks to promote overall health and disease prevention [12,13], offers a comprehensive approach to worker well-being. The project also draws on the health capital framework [14,15], to examine how individual and sociocultural resources influence workers’ engagement with preventive measures. Grounded in implementation science, the project also focuses on identifying key barriers and facilitators to intervention uptake across diverse occupational settings, assessing real-world feasibility, acceptability, and fidelity of intervention delivery [16].

This work introduces the CPW research project, detailing its methodological approach, intervention strategies, and evaluation metrics. It examines the potential of occupational health settings as a cost-effective and scalable platform for infection-related cancer prevention and the implementation of workplace-based interventions.

## 2. CPW consortium overview and work plan

The CPW Consortium comprises 18 partners and one associate partner from seven European countries – Italy, Spain, Romania, Slovakia, Denmark, Germany, and the Czech Republic **-**Table 1, combining expertise from academic institutions, public health authorities, healthcare providers, and industry partners. The CPW project is structured around pilot interventions aimed at preventing infection-related cancers within existing OHS programs. The project is implemented in various centers across four countries—Italy, Romania, Slovakia, and Spain —selected for their high incidence of the cancers associated with infectious agents [17,18].

**Table 1:** Work package leadership, implementing centers, and targeted worker groups.

Work Package (WP)	Focus Area	Leadership Country	Location of Implementing Centers	Targeted Worker Groups
**WP1**	Coordination and Data Management	Italy	–	–
**WP2**	Gastric Cancer Prevention (Hp Screening & Treatment)	Spain	Spain, Romania, Italy	Manufacturing, Healthcare, Retail
**WP3**	Liver Cancer Prevention (HCV Screening & Treatment)	Romania	Romania, Slovakia, Italy	Healthcare, Banking Sector, Retail
**WP4**	HPV-Related Cancer Prevention (Education, Awareness, and Vaccination)	Slovakia	Slovakia, Italy	Healthcare, Metal Manufacturing, Banking Sector
**WP5**	Behavioral and Sociocultural Assessment	Denmark	N/A	N/A
**WP6**	Cost-Effectiveness Analysis	Czech Republic	N/A	N/A
**WP7**	Dissemination, Outreach, and Exploitation	Spain	N/A	N/A
**Implementation Science**	Implementation research and scalability	Germany	N/A	N/A

Three key interventions are piloted: Hp screening and treatment, HCV screening and treatment, and HPV education and vaccination promotion. These initiatives target not only workers undergoing medical surveillance but also their family members. Mainly, workers testing positive for Hp or HCV are referred to the national healthcare system for diagnosis confirmation and treatment, in accordance with local clinical pathways. They also receive structured counseling to support family-based testing and increase awareness of infection risks within households. Each participating center implements a tailored combination of interventions to address the specific needs of its workforce, targeting various occupational groups such as healthcare workers, metalworkers, financial and service sector employees, and manufacturing workers Table 1.

**Fig 1:**
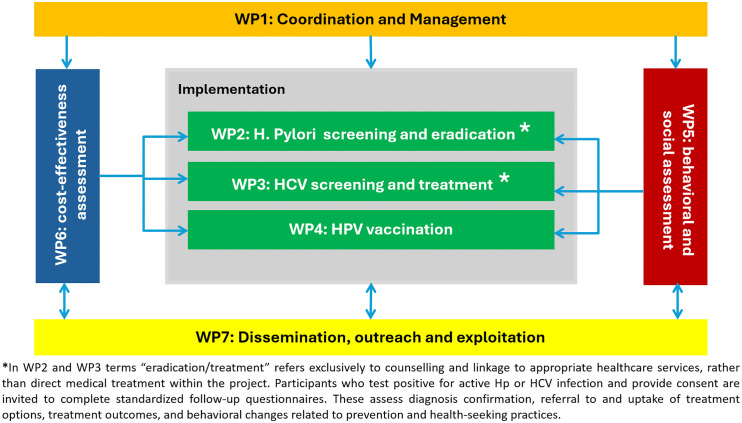
Overview of the CPW project structure and work packages.

The CPW project is organized into seven interlinked work packages (WPs) Fig 1, each covering a strategic component: project coordination and data management (WP1); pilot implementation of Hp, HCV, and HPV interventions (WPs 2–4). Two cross-cutting WPs complement these efforts: WP5 focuses on the sociocultural and behavioral barriers and facilitators that influence preventive interventions. WP6 conducts a cost-effectiveness analysis to guide future decision making. Additionally, WP7 leads dissemination, stakeholder engagement, and policy translation, ensuring that findings are effectively communicated and scaled for broader adoption. Importantly, the project integrates principles of implementation science across the pilot WPs to identify barriers and facilitators, inform adaptation strategies, and guide scale-up efforts. The objectives and tasks of each WP are briefly summarized in S1 Table. The work plan follows a phased approach—from preparatory activities (ethical approvals, protocol development, questionnaire harmonization) to the implementation of pilots with baseline data collection, followed by follow-up and final evaluation phases. The CPW project is guided by six specific objectives (SOs), each linked to defined expected outcomes (EOs) and measurable key performance indicators (KPIs) (Table 2). The selection of KPIs was informed by the project’s logic model and by frameworks for implementation and outcome evaluation consistent with Horizon Europe impact assessment principles. The KPIs capture three complementary dimensions of progress: process (e.g., number of finalized protocols, stakeholder engagement—O1, O5), output (e.g., participation and retention rates, workers surveyed—O2, O4), and outcome indicators (e.g., cost-effectiveness, scalability, dissemination reach—O3, O6). This structure aligns with the project’s implementation logic, linking operational delivery, behavioral insights, and policy translation within a coherent monitoring and evaluation framework. These objectives ensure a structured and outcome-driven approach to implementing and evaluating workplace-based cancer prevention interventions. By leveraging the collective expertise of consortium partners and aligning objectives with measurable outcomes, the CPW aims to establish a sustainable and replicable model for reducing the burden of infection-related cancers through proactive workplace engagement, thereby contributing to broader public health efforts in Europe.

**Table 2:** Study objectives and key performance indicators.

Objective	Expected Outcome	Key Performance Indicators
**O1.** Develop structured protocols for implementing cancer prevention initiatives within occupational health surveillance programs.	**EO1.** Established protocols for executing WP2-WP4 interventions in participating countries.	**KPI1.** Number of finalized protocols and pilot studies.
**O2.** Implement and evaluate cancer prevention interventions (WP2-WP4) among targeted worker populations, integrating prevention strategies, behavioral insights, and implementation science.	**EO2.** Completion of workplace-based cancer prevention interventions in line with standardized protocols.	**KPI2.** Total number of workers participating; participation and retention rates.
**O3.** Assess the cost-effectiveness of the interventions conducted under O2, including the potential for broader application.	**EO3.** Comprehensive cost-effectiveness evaluation of interventions, including feasibility for scaling up to additional sectors or countries.	**KPI3.** Net cost assessment and potential savings; identification of key factors affecting cost-effectiveness.
**O4.** Investigate behavioral and sociocultural factors influencing participation in workplace cancer prevention programs.	**EO4.** Identification of key facilitators and barriers, along with actionable recommendations to optimize intervention uptake.	**KPI4.** Number of workers surveyed or interviewed.
**O5.** Strengthen engagement with public health authorities and key stakeholders to support the integration of cancer prevention measures in workplace settings.	**EO5.** Development of a strategic implementation plan with contributions from relevant stakeholders.	**KPI5.** Number of public health authorities involved in the initiative.
**O6.** Disseminate project findings and encourage broader adoption of workplace-driven cancer prevention strategies.	**EO6.** Implementation of a communication and dissemination plan targeting scientific communities, policymakers, and the general public.	**KPI6.** Number of conference attendees, public data users, and citations of scientific publications.

### Work Package 1: Coordination and management

WP1 serves as the central hub for governance, risk management, and administrative functions, ensuring effective coordination across the consortium. It is responsible for overseeing the scientific and administrative management of the CPW project, with a focus on adherence to ethical, regulatory, and financial standards. WP1 plays a key role in coordinating the data collection process, particularly during the questionnaire conceptualization phase, ensuring harmonization across the participating implementation centers. This coordination also includes the development of materials centrally, reducing duplication of effort and ensuring consistency in approach across the intervention WPs. Furthermore, WP1 is responsible for training personnel involved in data collection, ensuring they are proficient in using electronic tools for storing data in a centralized repository. By facilitating coordination and supporting a unified approach, WP1 helps maximize the effectiveness of the overall intervention, enhances the likelihood of a consistent, scalable methodology, and allows for aggregated conclusions that are essential to the study’s overall success. WP1 also ensures that data management and statistical analysis comply with ethical standards, maintain data security, and adhere to FAIR (Findable, Accessible, Interoperable, Reusable) principles, making the project’s data usable beyond the project duration.

### Work Package 2: Gastric cancer prevention through *Helicobacter pylori* screening and treatment

WP2 designs and implements a protocol for HP screening, targeting workers involved in OHS and extending screening recommendations to family members of HP-positive workers. Hp infection is a leading risk factor for gastric cancer, and early detection through screening and eradication is a key strategy for reducing cancer incidence [19]. Despite the success of Hp test-and-treat programs in high-risk countries, workplace settings have remained underexplored in this context [13,20]. Therefore, the WP2 approach leverages the workplace setting to identify infected individuals and promote preventive action before the onset of disease.

WP2 coordinates and implements pilot projects in several CPW centers, focusing on identifying Hp infection rates among workers and evaluating the effectiveness of screening protocols. Data collected from these projects support the analysis of determinants of Hp infection and assess the feasibility and acceptability of the intervention. Based on these findings, the work package develops plans for the large-scale implementation of Hp prevention within occupational health surveillance systems. Although medical treatment of Hp lies outside the scope of the project, WP2 provides all workers with a comprehensive counseling package. This includes evidence-based information on Hp-related cancer risks, integrated diagnosis and treatment options, and available care pathways within their local healthcare systems. Workers who test positive for Hp also receive guidance on encouraging screening among their family/household members, who are considered at increased risk. At least six months after the intervention, positive participants undergo follow-up according to a protocol specific to each intervention, using standardized questionnaires to assess diagnosis confirmation, treatment uptake and outcomes, attitudes toward the intervention, and behavioral changes.

Furthermore, national and regional stakeholders engage in the adaptation of country-specific preventive interventions tailored to local practices in occupational medicine, ensuring the integration of these interventions into broader public health strategies.

### Work Package 3: Liver cancer prevention through HCV screening and treatment

WP3 focuses on the prevention of liver cancer through the screening and treatment of Hepatitis C Virus (HCV) infection in occupational settings. In Europe, chronic HCV infection is a leading cause of liver cirrhosis, which can progress to liver cancer, making early detection and treatment crucial [21]. This work package aims to design and implement a protocol for HCV screening, targeting workers involved in occupational health surveillance and extending the screening to family members of HCV-positive workers. As a key strategy in the fight against liver cancer, this approach takes advantage of the unique opportunity provided by the workplace setting to identify infected individuals and to offer access to treatment. In this process, the occupational health doctor plays the role of a healthcare navigator, guiding employees and their household members inside the healthcare system and helping them to understand the diagnosis and treatment plan. The work package involves the coordination and implementation of pilot projects at several CPW centers, focusing on identifying HCV infection rates among workers and evaluating the effectiveness of the screening protocols. The testing sequence for identifying current HCV follows the updated WHO’s recommendation and starts with serological testing (rapid diagnostic test or laboratory-based immunoassay) offered to all eligible workers [22]. Before starting the pilot, the occupational health staff received training on the standard protocol and guidance on how to provide outreach and education to raise participants’ awareness about the HCV risks and behavioral changes. Health education and promotion campaigns (newsletters, promotion materials, face-to-face discussions) were developed to be culturally appropriate for each targeted occupational group. Data collected from these projects are analyzed to understand the determinants of HCV infection and to assess the success of the intervention. Based on the findings, plans will be developed for the large-scale implementation of HCV prevention within occupational health surveillance systems. Furthermore, national and regional stakeholders are engaged in adaptating country-specific preventive interventions tailored to local practices in occupational medicine, ensuring the integration of these interventions into broader public health strategies for liver cancer prevention.

### Work Package 4: Prevention of cancers associated with HPV infection

WP4 aims to prevent cancers associated with HPV infection through education, increasing awareness, and vaccination. HPV infection is linked to several types of cancers, located in the anogenital and head and neck regions, most notably cervical and oropharyngeal cancers [23]. Vaccination against high-risk HPV types can significantly reduce the incidence of these cancers if given before exposure to HPV infection [24–26]. This work package seeks to implement a strategy that targets both workers and their eligible family members for HPV vaccination. WP4 provides all workers with a comprehensive counseling package that includes information on HPV-related cancer risks, behavioral factors, and the integrated diagnosis and care pathways available in their local communities. An important part of the project is the intervention aimed at training and further education of occupational health service teams who carry out preventive medical examinations at work. The primary objective of this activity is to ensure successful uptake and response to the wider prevention of cancer diseases that are not directly related to occupational risks. The emphasis is on personal communication, guidance, training, and counseling services provided by principal investigators in each center under WP4 coordination. Data from these pilot projects are analyzed to identify the key determinants and indicators of vaccination uptake as well as the effectiveness of the intervention. Based on the results, WP4 develops a plan for the large-scale implementation of HPV vaccination within occupational health surveillance systems across Europe. Furthermore, national stakeholders are engaged in adapting existing preventive interventions and implementing tailored HPV vaccination programs in accordance with local needs and public health policies. This work package aims to strengthen the integration of primary HPV prevention into workplace health programs, thereby improving overall vaccination rates and reducing the long-term burden of HPV-related cancers.

### Work Package 5: Behavioural and sociocultural assessment

WP5 aims to understand the sociocultural and behavioral factors that influence participation in primary prevention interventions for infection-related cancers. By identifying and assessing barriers and facilitators to screening uptake, vaccine hesitancy, and patient pathways after positive screening results, this work package provides critical insights for future optimization of the effectiveness of the interventions developed in WP2–WP4. To achieve this, the first phase of WP5 involves a mixed-methods study that combines secondary data from the literature with primary qualitative and quantitative data collected through baseline questionnaires, projective techniques, and focus groups. The data provides insights into how workers relate their occupation and their health, their perception of the relative importance of health-related resources at their disposal, and their understanding of the screening, treatment, and vaccination processes. The 12-Circles Drawing Technique employed in the first phase is a projective technique targetedly designed for the CPW project, allowing to collect data from contextually diverse worker cohorts [27]. Data are first analyzed inductively, followed by a deductive comparison with existing models. To ensure validity and reliability, all qualitative data will be analyzed by at least two researchers. Building on this, in the second phase, WP5 develops the Health Capital Questionnaire (HCQ) to quantify the barriers and facilitators of preventive interventions against Hp, HPV, and HCV. The questionnaire structure will be informed by findings from the first phase. The HCQ assesses individual, social, cultural, and economic factors affecting adherence and participation. In a large-scale survey study, the health capital framework [14,15] is used to capture how workers’ personal and social resources—such as health knowledge, social networks, and financial means—shape their decisions regarding preventive measures.

By integrating insights from both phases, WP5 provides evidence-based recommendations for tailoring prevention programs to better address sociocultural and behavioral factors, ultimately enhancing intervention accessibility, improving participation rates, and supporting the scalability of prevention strategies across diverse occupational settings.

### Work Package 6: Cost-effectiveness analysis

Work Package 6 focuses on the assessment of the cost-effectiveness of the interventions piloted under WPs 2–4. This includes the comparison of direct intervention costs (e.g., screening), indirect costs (e.g., treatment), and the benefits generated as a result of the interventions (avoided costs of ill health). CEA also aims to identify and evaluate the key factors that influence the economic outcomes of these interventions, including the design of the intervention and the structural conditions, such as the characteristics of the target populations or framework conditions in the relevant country. Another key objective is to assess the scalability of the interventions, examining whether they can be extended to other industries and EU Member States. This analysis also aims to contribute to the identification of the conditions under which scaling up similar interventions could be both cost-effective and sustainable. While previous studies suggest that screening and eradication programs for Hp, HCV, and HPV vaccination have the potential to be cost-effective, WP6 aimes to improve the understanding of the extent to which interventions targeting the three cancer-related infections implemented within the framework of OHS in Europe can be cost-effective. The ultimate goal is to build a more robust evidence base for the cost-effectiveness of workplace-based public health interventions.

### Work Package 7: Dissemination, outreach, and exploitation

Work Package 7 focuses on maximizing the impact of the project through effective communication, dissemination, and strategic engagement with stakeholders. The goal is to ensure that the project’s results are widely shared and embedded in systems that enable long-term sustainability and implementation. The objectives of WP7 include developing a dynamic and evolving plan (CDE Plan) for disseminating the project’s results, engaging stakeholders to facilitate broader implementation, and piloting a family-centered engagement approach to assess its impact on prevention adherence. Additionally, WP7 manages intellectual property rights (IPR) and develops a strategy to ensure the long-term exploitation of project results. WP7 works across the entire project, coordinating with the consortium, intervention centers, and partners to ensure coherent and impactful communication. The CDE Plan has defined target audiences, communication tools, and actions in order to ensure that the project’s messages reach the right stakeholders. This includes a project website, newsletters, social media, educational videos, scientific publications, and other educational formats targeting general audiences, such as scientific monologues. The plan also aims to engage stakeholders through events, workshops, and partnerships, with a special focus on multi-level engagement –from local implementation sites to EU-wide policymaking forums. In close collaboration with WP2, WP3, and WP4, WP7 also supports the development of health education and promotion campaigns tailored to local contexts. This includes study brochures and promotional materials developed in multiple languages, adapted to different occupational groups at intervention sites, ensuring accessibility and relevance across all participating countries. WP7 also actively contributes to the “Prevention and Early Detection Implementation research” cluster, strengthening the project’s policy relevance and alignment with broader European cancer prevention strategies. In addition, WP7 plays a central role in translating evidence into actionable policy. The work package coordinates the preparation of the CPW Green Paper and targeted policy briefs, synthesizing findings from all WPs into practical recommendations for EU and national decision-makers. Ultimately, WP7 ensures that the project’s results reach a wide audience, influence policy, and contribute to the long-term success of the interventions. Dissemination activities are continuously monitored and adapted based on performance indicators, ensuring the sustainability and scalability of the interventions in both the short and long term.

### Implementation science

Implementation science plays a crucial role in ensuring the successful large-scale adoption of the CPW project’s workplace-based interventions, and its activities are included under the task of WP2–4. Among tasks of WP2–4, frameworks for implementation science are employed to analyze the factors that influence the uptake of the interventions, based on data gathered in WP2–6. Employers should commit to investing in cancer prevention for their workforce, which can be challenging, especially if the procedures extend beyond strictly work-related risks. The organizational arrangements for occupational healthcare professionals vary within and across countries [28], ranging from integration within organizations to operating as independent service professionals. Certain organizational characteristics, such as stability and turnover rates among occupational healthcare professionals, can influence the probability of implementing new practices. In some countries, mandatory procedures have been established within occupational health services, while in others, these procedures are recommended rather than required. Some but not all companies offer medical examinations and health promotion services that address issues beyond occupational risks [29]. In addition, the qualifications and attitudes of individual occupational healthcare providers, who are responsible for delivering preventive interventions, are crucial for implementation. Factors such as their competence in providing these interventions, their acceptance of delivering services that extend beyond work-related risks, and their perceptions of the feasibility of such adoption all play a role. Occupational healthcare providers involved in cancer prevention and new procedures require specific competencies and should be provided with appropriate diagnostic tools (e.g., blood samples and stool antigen tests for Hp screening, blood samples for HCV) and preventive tools – vaccines (anti-HPV). Additionally, medical laboratories require sufficient qualified staff to analyse these tests effectively (e.g., Hp and HCV). The CPW project systematically documents the perceptions of occupational healthcare providers regarding potential barriers and facilitators to implementing three occupational-based interventions. The Consolidated Framework for Implementation Research (CFIR), which identifies potential barriers and facilitators across five domains, serves as a guiding tool for this analysis [30].

The success of prevention programmes as a part of occupational health surveillance arguably requires acknowledging and embracing worker diversity. Across different occupational cohorts, the diversity of workers’ educational and cultural backgrounds, socioeconomic resources, and behavioural patterns comprising their health capital requires the targeted adaptation of preventive interventions with a view to better implementation in practice [15]. Even within one occupational cohort, sociocultural and behavioural barriers to and facilitators of cancer prevention challenge a one-size-fits-all approach [3]. The CPW project investigates and assesses patterns of inter- and intra-cohort diversity using projective techniques for self-assessment of the relative importance of contextual factors for workers’ health. This projective technique doubles as a personalized small-scale intervention, encouraging workers to reflect on the importance of their health and the resources that they possess that might be used to nurture their health and well-being. Moreover, explorative interview research with healthcare providers further uncovers insights into facilitators and barriers to the implementation process. These findings inform structured group meetings aimed at providing recommendations for the sustainable scaling-up of the interventions. Identifying both strengths and weaknesses in the user organizations’ capacity to adopt new health interventions is critical for the successful expansion of these programs across diverse EU countries. The comprehensive process evaluation ensures that implementation strategies are tailored to local contexts, thereby maximizing the interventions’ effectiveness and sustainability in occupational health settings.

## 3. Methods: Data collection strategy

### Design, setting, and sample size

The study is conducted using a prospective approach across multiple research sites in Italy, Spain, Romania, and Slovakia, involving different groups of workers. Table 1 summarizes details about the study partners, interventions, and worker populations. The data collection is conducted in two phases, at baseline and during follow-up. All workers participating in the Occupational Health Surveillance (OHS) are approached either before or during their routine occupational health check-ups and are invited to participate in the CPW project. Participant enrolment is planned for a period of 24 months, from month 13 to month 36 of the project duration, with follow-up activities continuing through to month 42. While some methodological details may differ between interventions, the overall project adheres to common approaches.

### Inclusion and exclusion criteria

Occupational health professionals assess eligibility criteria and exclusion criteria for each specific intervention. The inclusion and exclusion criteria for each intervention are summarized in S2 Table.

### Recruitment and informed consent

Informative materials explaining the purpose of the study are distributed at the workplace in local languages to enhance understanding of the study objectives and encourage acceptance of the study procedures. In the days leading up to their OHS visit, workers receive an invitation to participate in the study. Participation is entirely voluntary and enrollment takes place during regular OHS visits. At the time of the visit, occupational health professionals, such as physicians or nurses, provide further detailed explanations about the project and invite the worker to complete a written informed consent form.

### Materials, procedures, interventions, and outcome measures

#### Baseline phase.

A baseline questionnaire is administered to consenting workers by trained interviewers from the OHS staff in a pseudo-anonymized format. These questionnaires are completed on-site to ensure the privacy of respondents. The self-administered sections of the baseline questionnaire are filled out on paper and collected in secure, closed boxes or envelopes. A subset of participants from selected centers also engages in a self-administered projective technique to evaluate their perceived importance of sociocultural and economic resources for health, which are covered by WP5. In some centers, enrolment may also occur via telephone, depending on the circumstances. The collected data is initially on paper questionnaires, which are then transported to electronic software (REDCap) or entered directly into the software during recruitment. Additional related biosamples are also collected. Details of the recruitment strategy for each work package are summarized in Table 3.

**Table 3:** Summary of frameworks and intervention strategies for each work package.

Intervention type/working package	Baseline questionnaire	Serology test/additional intervention	Strategy with positive infections	Follow up
**Working packages with intervention**				
**WP2-HP**	• Informed consent form • questionnaire screening test	• Blood Inmunoglobulins G, A, M Test • Stool antigen test (SAT) for positive serology	• Proposal for eradication by the healthcare system • Refer household members to the health care system	6-month follow-up, including a questionnaire based on the positivity of SAT (Stool Antigen Test)
**WP3-HCV**	• Informed consent form • questionnaire screening test	• HCV rapid test • laboratory based immunoassay (EIA)	• Proposal of treatment • invitation to screen household members	6-month follow-up, including a questionnaire
**WP4-HPV**	• Informed consent form • questionnaire screening test	• Vaccination • Invitation to household members to undergo vaccination	N/A	6-month follow-up, including a questionnaire
**Working packages without intervention**				
**WP5- Behavioural and Sociocultural assessment**	Phase 1: Participants for the projective techniques and focus groups are recruited from a subset of the cohorts recruited for WP2–4. Phase 2: All cohorts from all implementation centers comprise the participants for the large-scale survey study based on the HCQ.			
**WP6- Cost-effectiveness analysis**	The estimation of the costs follows a methodology comprising primary data collection for the CPW interventions and their combination with published data for the costs incurred by public healthcare systems for patients and FMs referred by CPW interventions.			
**Implementation Science**	Baseline and follow-up questionnaires for occupational healthcare providers delivering the intervention in each implementing center in WP2–4.			

#### Follow-up phase.

At least six months after the intervention, participants **are followed up** according to intervention-specific protocols using standardized questionnaires. These questionnaires aim to assess diagnosis confirmation, treatment progress and outcomes, attitudes toward the intervention, and any related behavioral changes. In WP2 and WP3, only participants who tested positive with active infection for Hp or HCV and consented to follow-up **are invited** to complete the questionnaires. In WP4, all participants who express a willingness to be vaccinated **are asked** to complete a follow-up questionnaire to evaluate their final attitudes toward HPV vaccination and any resulting behavioral changes.

#### Household members.

The OHS approach, in addition to assisting workers, can also support other health promotion initiatives that benefit other population groups, particularly the workers’ household members. In this project, household members are engaged indirectly in different ways across the interventions to extend cancer prevention beyond the workplace. In the Hp intervention, workers who test positive receive counseling and recommendations for screening of their household members, emphasizing the potential transmission risk. Positive workers are referred to their general practitioner (GP) or other healthcare provider for further management. Those with confirmed active infections are contacted for a follow-up questionnaire, which collects limited information regarding their care pathway and treatment, and family members’ screening. In the HCV intervention, workers who test positive are advised about the importance of screening their household members through available local medical services. In the HPV intervention, workers whose household members meet the eligibility criteria are invited to facilitate their participation. Eligible household members are offered vaccination through the workers. Vaccinated workers are followed up after around six months, with an assessment of the vaccination status of their household members.

#### Questionnaire and list of variables.

This study involves two main sets of questionnaires: baseline questionnaires and follow-up questionnaires. Each questionnaire contains common sections applicable to all interventions, as well as specific questions related to each infection-specific intervention (HP, HCV, and HPV). The key areas of analysis include sociodemographic information, occupational history, diet and nutrition, medical history, family history, self-perceived health, emotional stress, risk behaviors, risk factors associated with each infection, and knowledge of the infections and preventive strategies. Participants undergoing HCV screening are asked to self-report sensitive information regarding personal habits, such as sexual practices and drug use, which are considered risk factors. During the design of the questionnaires, we used different international sources such as the International Classification of Diseases, 3rd Edition (ICD-O-3) [31]. A detailed list of variables is provided in S3 Table.

### Data management plan and safety considerations

Data collection and analysis are conducted on the AlmaHealthDB (AHDB) infrastructure – a technological and organizational infrastructure supporting the management of large volumes of research data. AHDB ensures compliance with legal, ethical, organizational, and regulatory requirements. It consists of a series of virtual machines and a private network hosted within the Emilia-Romagna Regional Health Service network. The infrastructure can be used for both single-center and multi-center studies. For each individual research project, dedicated virtual machines are provisioned within the “trust” area of the infrastructure. These can securely connect to virtual machines with a public IP, exposing the data ingestion services.

Within the CPW project, REDCap (Research Electronic Data Capture) was employed as a data ingestion service to produce structured data collected through Electronic Case Report Forms (eCRFs) and surveys [32,33]. Based on a master project, center-specific versions were derived and translated into the local language of each site, thus developing separate REDCap projects for each implementation center. This approach ensures both regulatory compliance – by restricting access to data entered by each center only – and harmonization of data across the consortium. Once the data collection is complete, data can be shared in accordance with pre-defined access policies. Given the sensitive nature of the data generated during the CPW project, specific policies on open access are implemented to ensure ethical and legal compliance. In particular, data sharing occurs only after proper anonymisation procedures have been applied. Project participants report any restrictions on data access. By the end of the project, all datasets become available in the Observational Medical Outcomes Partnership (OMOP) Common Data Model and are described using OMOP standard metadata [34].

### Quality assurance and control

To ensure data completeness and validity of the collected data, our team adopted a comprehensive approach throughout the questionnaire development and validation phases. A preliminary harmonization process was carried out to align the questionnaires across the different WPs. This involved the development of a centralized CPW Master project in REDCap, which served as the foundational template. The Master project was then duplicated and tailored to reflect the specific requirements of each CPW center, based on their WP involvement. Translations in the local language were subsequently applied. Throughout the development process, a range of validation controls were embedded into the REDCap system, including constraints on data types, acceptable value ranges for numerical fields, and mandatory fields that trigger error messages if left incomplete

Prior to deploying the questionnaires in production, several quality assurance steps were implemented: 1) each implementation center was granted access to a development version of their tailored questionnaire to conduct preliminary testing; 2) live demonstration sessions were held with the centers to collaboratively identify and resolve issues; 3) the questionnaires were finalized following centers’ feedback; 4) a final revision was conducted by the respective center. Once approved, the REDCap projects were migrated to production, where actual data collection started.

In addition to the technical controls, further measures were taken to enhance data quality: 1) training sessions were conducted with all data collectors to clarify procedures and minimize misunderstandings; 2) a sample of completed questionnaires from each center was systematically reviewed; 3) software-based cross-validation rules were applied to detect missing data and to prevent the inclusion of implausible or out-of-range responses.

### Statistical analysis

The primary outcomes focus on process indicators for the interventions, including: (i) participation rate of occupational health professionals, (ii) acceptance rate among workers, (iii) completion rate of screening test for Helicobacter pylori (Hp) and hepatitis C virus (HCV), as well as HPV vaccination, (iv) positivity rate for Hp and HCV in the project sample size (v) treatment acceptance rate for Hp and HCV (i.e., participants who initiated treatment), (vi) treatment completion rate for Hp and HCV (i.e., participants who completed treatment), (vii) rate of health issues associated with Hp and HCV treatment, and (viii) household member acceptance of HPV vaccination. Secondary outcomes address detailed aspects of the intervention, such as the participation of GPs in the second step of the intervention on Hp. Analyses are conducted center-wise with a standardized approach. Initially, a descriptive univariate analysis is performed, stratified by age, sex, and job title. This is followed by multivariate models to identify outcome determinants, employing logistic regression for dichotomous variables and generalized linear models for continuous outcomes. Heterogeneity is assessed using the Q-test, and if p < 0.01, random-effects meta-analyses are used to combine results. Statistical analysis is conducted using different functions developed within the STATA software package.

### Timeline and study status

Enrollment started in May 2024- January 2025 and is expected to continue for approximately 24 months (estimated by April 2026 or until the target number of participants is reached). Data collection, including all follow-up assessments, is expected to conclude by October 2026. The final study results are anticipated by the end of April 2027, in alignment with the project’s planned end date.

### Ethical considerations

The study has been approved by the independent local ethics committees at each implementation center. For more details, please refer to the Ethics Statement section. Specifically, approvals were obtained from the following bodies: Comitato di Bioetica Alma Mater Studiorum Università di Bologna (29 February 2024), Comitato di Bioetica d’Ateneo Università di Torino (19 March 2024), Comisia de Etică a Cercetării din Spitalul Colentina (26 February 2024), Timisoara Municipal Emergency Clinical Hospital Manager (19 January 2024), Etická Komisia FNsP F.D. Roosevelta Banská Bystrica (27 February 2024), Advisory Group of the Regional Hygienist RAPH BB (5 March 2024), Nezávislá Etická Komisia Banskobystrického (21 May 2024), and Comité de Ética de la Investigación del Principado de Asturias (14 December 2023).

## 4. Discussion

The CPW project is anticipated to provide strong evidence on the feasibility, cost-effectiveness, and acceptability of cancer prevention interventions within OHS. By targeting infection-related cancers—gastric, liver, cervical, anogenital, and oropharyngeal cancers—the project is positioned to make a substantial public health impact, reducing the incidence and mortality of these preventable diseases. The project’s multifaceted approach, which involves both workers and their household members, extends its impact to the broader population, potentially benefiting a significant portion of society.

The CPW project’s integration with OHS programs is one of its greatest strengths. The established relationships between occupational health professionals (OHP) and workers provide a strong foundation for implementing interventions, enhancing their likelihood of success. Additionally, the active engagement of public health authorities in each implementation center is a project’s strength. The effectiveness of workplace interventions in promoting cancer screening has been documented in recent studies. A systematic review highlighted that such interventions can positively influence cancer screening uptake among employees, leading to earlier detection of breast, colorectal, and cervical cancers [7]. The review emphasized the importance of designing evidence-based, structured interventions in collaboration with researchers, public health specialists, and healthcare systems to ensure their sustainability and effectiveness [7]. The implementation of the CPW project may face challenges related to data collection and heterogeneity in implementation approaches across different countries. This heterogeneity arises from both study-related factors—such as the use of different screening tools, primary data collection methods, and the complexity of interventions (e.g., single vs. multi-component)—and non-study-related factors, including variations in OHS structures, resource availability (e.g., staffing levels, equipment), legal frameworks, and the extent of integration with national health systems [35,36].

Additional factors that may affect data quality include confidentiality concerns, workforce diversity, and regional discrepancies. These discrepancies may involve differences in health literacy, socioeconomic conditions, urban versus rural access to health services, language or dialect use, and regional disease profiles—all of which can impact participation, reporting accuracy, and the feasibility of applying standardized interventions across settings [37–40]. Building on these contextual factors, the CPW project explicitly incorporates an equity perspective by considering gender, socioeconomic background, and cultural or linguistic diversity as key determinants of participation and access. Although migrant status is not directly recorded, questionnaires include items on ethnicity, language proficiency, and perceived cultural belonging, allowing indirect analysis of equity-related differences across populations. This ensures that prevention strategies are sensitive to workforce diversity. Furthermore, public health surveillance poses ethical challenges—such as the risk of collecting incomplete or inaccurate data and the potential stigmatization of specific subgroups—which must be carefully addressed to preserve both the scientific integrity and the social acceptability of the project [41,42]. Overcoming these challenges requires addressing both logistical and cultural barriers. The CPW project aims to mitigate these obstacles by leveraging existing OHS infrastructures, establishing a centralized data management unit, and using platforms like REDCap to streamline data ingestion and analysis, thereby improving overall efficiency and data reliability [33,43]. In CPW, each intervention is tailored to the specific needs of occupational groups, with the project’s cross-country scope strengthening the generalizability of its results across Europe. The interventions will target workers and their families, amplifying the impact on public health. Family-centred health promotion emphasizes families’ critical role in maintaining health and preventing disease within communities [44]. Family-inclusive well-being programs foster a supportive environment that reinforces healthy behaviors both at work and at home [44,45]. Therefore, by extending health interventions beyond the workplace and into employees’ households, organizations can maximize the long-term impact of their health promotion efforts, ultimately contributing to broader public health benefits.

Through the assessment of sociocultural and behavioral barriers and facilitators of interventions for infection-related cancers, the project contributes to the broader understanding of patterns of (non-)participation in screening, vaccination, and treatment processes. Based on this understanding, the consortium is expected to formulate recommendations for addressing barriers and exploiting facilitators of preventing infection-related cancers in the context of OHS. A recent systematic literature review showed that sociocultural and economic barriers have also been identified in cancer prevention efforts, where participation in vaccination and screening programs is shaped by factors such as access to healthcare, health literacy, and cultural beliefs [3]. While scaling similar interventions beyond the pilot phase may be challenging due to diverse healthcare infrastructures across countries and potential resistance in certain regions, particularly in low- and medium-income countries, addressing sociocultural barriers to participation early on will be important. Understanding and overcoming these barriers is key to ensuring the long-term success and scalability of the project. Furthermore, the involvement of a diverse range of stakeholders, including public health authorities, professional associations, and non-governmental organizations, ensures the sustainability of the intervention and fosters broad ownership of the project’s objectives, increasing the likelihood of long-term success. This multi-actor approach is supported by a structured Stakeholder Engagement Plan and a dedicated forum that channels ongoing input into the design, implementation, and policy relevance of the interventions. The diversity enables the project to address implementation challenges from multiple angles—scientific, institutional, social, and regulatory—making the proposed interventions more adaptable and resilient across contexts. The cross-country implementation and focus on various occupational groups contribute to the scalability and applicability of the interventions across Europe. The diversity of sectors and industries involved, coupled with a coordinated dissemination and engagement strategy, enhances the potential to replicate these interventions beyond the pilot phase and support their integration into occupational health systems at national and EU levels. Lessons learned from this project could serve as a model for expanding workplace-driven cancer prevention initiatives to other settings, including low- and middle-income countries facing similar public health challenges. By creating a scalable framework, the CPW project has the potential to influence cancer prevention policies and practices on a larger scale.

## 5. Conclusions

The CPW project exemplifies a transformative approach to cancer prevention by leveraging the untapped potential of OHS. Its focus on infection-related cancers addresses a critical gap in cancer prevention efforts, while its innovative use of data management and collaborative frameworks ensures that the interventions are both effective and scalable. By aligning with EU priorities and emphasizing stakeholder engagement, the CPW project not only advances the science of cancer prevention but also sets a precedent for integrating public health initiatives into occupational settings. This manuscript provides an overview of the CPW project’s objectives, methodologies, and anticipated impact, highlighting its contribution to the global fight against cancer. A unique aspect of the CPW project is its emphasis on workforce health as a lever for broader societal impact. By improving the health of workers, the project indirectly benefits their families and communities, creating a ripple effect that extends beyond the workplace. This aligns with the EU’s goals of promoting equity and sustainability in healthcare, contributing to the larger mission of the Horizon Europe program to advance implementation science for cancer prevention.

## Supporting information

S1 Table WP’s objectives/tasks summary. (DOCX)

S2 Table Inclusion and exclusion criteria for HP, HCV, and HPV interventions. (DOCX)

S3 Table Details of baseline and follow-up questionnaires for HP, HCV, and HPV interventions. (DOCX)

## Abbreviations

CEA: Cost-Effectiveness Analysis
CFIR: Consolidated Framework for Implementation Research
CPW: Cancer Prevention at Work
eCRFs: Electronic Case Report Forms
FAIR: Findable, Accessible, Interoperable, Reusable
HCV: Hepatitis C Virus
HCQ: Health Capital Questionnaire
Hp: Helicobacter pylori
HPV: Human Papillomavirus
ICD-O-3: The International Classification of Diseases, 3rd Edition
IPR: Intellectual Property Rights
KPIs: Key Performance Indicators
OHS: Occupational Health Surveillance
OMOP: Observational Medical Outcomes Partnership
ORs: Expected Outcomes
REDCap: Research Electronic Data Capture
SOs: Specific Objectives
TWH: Total Worker Health
WPs: Work Packages
