# Supplementary material for: Cancer Prevention at Work (CPW) project: Rationale, framework and research protocol
Source: PLoS One. 2025 Nov 3;20(11):e0335752. doi: 10.1371/journal.pone.0335752 (PMC12582473; doi:10.1371/journal.pone.0335752)
Supplement: S1 Table — (DOCX) [file pone.0335752.s001.docx]

**S1 Table. WP's Objectives/Tasks summary.**

| **Work Package** | **Objectives/Tasks** |
| --- | --- |
| **WP1: Coordination and Management** | - Monitor project progress against overall objectives, key milestones, and deliverables, ensuring smooth coordination across all WPs. |
|  | - Install all managerial bodies and procedures to oversee project execution. |
|  | - Ensure compliance with technical and financial reporting to the European Commission (EC) as per the EC Grant Agreement. |
|  | - Harmonize the development of activities across all WPs to ensure effective collaboration. |
|  | - Facilitate communication between project partners and the European Commission to ensure efficient project management and reporting. |
|  | - Maintain the CPW Consortium Agreement and oversee ethical, regulatory, and data management compliance. |
|  | - Provide support for statistical analysis of the data collected within pilot projects, ensuring scientific integrity. |
|  | - Develop and maintain the Dissemination and Exploitation Plan, ensuring effective data and knowledge management. |
| **WP2: Gastric Cancer Prevention** Through Helicobacter Pylori Screening and Treatment | - Design a protocol for Helicobacter Pylori (Hp) screening and treatment for workers involved in occupational health surveillance, including follow-up, referral to the health system and screening of family members of Hp-positive workers. |
|  | - Coordinate the implementation of the pilot project on Hp prevention at several centers participating in CPW. |
|  | - Analyze the data collected within the pilot projects on Hp prevention on determinants of Hp infection and effectiveness of intervention.. |
|  | - Based on the results of the pilot projects, to develop plans for the large-scale implementation of Hp prevention within occupational health surveillance systems and clinical and public health system.  - To collaborate with public health administration and community stakeholders to identify the best strategies to increase the population's adherence to the program of screening and eradication of HP at the community level in Europe. |
| **WP3: Liver Cancer Prevention Through HCV Screening and Treatment** | - Design a protocol for Hepatitis C Virus (HCV) screening and treatment for workers involved in occupational health surveillance, including family member screening and follow-up. |
|  | - Implement the pilot project for HCV prevention at multiple CPW centers and monitor the outcomes. |
|  | - Analyze pilot data to assess the impact of HCV prevention and treatment strategies and develop plans for large-scale implementation within national health systems. |
| **WP4: Prevention of Cancers Associated with HPV Infection** | - Design a protocol for HPV vaccination targeting workers involved in occupational health surveillance, with follow-up and vaccination for eligible family members. |
|  | - Implement the pilot HPV vaccination project across participating centers to assess vaccine uptake and effectiveness. |
|  | - Scale up the HPV vaccination strategy based on pilot results to integrate within national occupational health surveillance systems across Europe. |
| **WP5: Behavioural & Sociocultural Assessment** | - Identify barriers and facilitators related to behavioral and sociocultural factors that influence the effectiveness of infection-related cancer prevention interventions. |
|  | - Develop the Health Capital Questionnaire (HCQ) to assess these behavioral and sociocultural factors in the occupational cohorts. |
|  | - Quantify these factors through a large-scale survey and develop recommendations to address barriers and enhance the success of prevention programs. |
| **WP6: Cost Effectiveness Analysis** | - Assess the cost-effectiveness of the interventions compared to a no-intervention scenario and interventions targeted at the public. |
|  | - Evaluate the key factors influencing cost-effectiveness in the design and implementation of the interventions. |
|  | - Assess the scalability of the interventions to other sectors and EU member states, determining their potential for broader application. |
| **WP7: Dissemination, Outreach & Exploitation** | - Design a communication and dissemination plan to optimize knowledge transfer and engagement with relevant stakeholders. |
|  | - Engage stakeholders to ensure the broader implementation and sustainability of project results. |
|  | - Manage intellectual property (IP) and create a roadmap for the exploitation of project results, ensuring long-term impact and continued use of findings. |
|  | - Develop a family-centered engagement pilot at one participating center to evaluate its impact on adherence to prevention interventions. |
