# Supplementary material for: Cancer Prevention at Work (CPW) project: Rationale, framework and research protocol
Source: PLoS One. 2025 Nov 3;20(11):e0335752. doi: 10.1371/journal.pone.0335752 (PMC12582473; doi:10.1371/journal.pone.0335752)
Supplement: S2 Table — (DOCX) [file pone.0335752.s002.docx]

**S2 Table. Inclusion and exclusion criteria for HP, HCV, and HPV interventions.**

| **Intervention** | **List of inclusion and exclusion criteria** | |
| --- | --- | --- |
| **Hp** | Inclusion criteria | Workers undergoing OHS, Age: 25-65 |
|  | Exclusion criteria | Unable to provide informed consent, no means of obtaining adequate biological samples, diagnosed with Hp, and ongoing, or partially, or completed treatment, not able to undergo Hp therapy and eradication. Temporary exclusion criteria: Diarrhoea, Currently Antibiotics/Proton pump inhibitors/H2 blockers/antacids (stop 4 weeks) |
| **HCV** | Inclusion criteria | Workers undergoing OHS, Age: >18 |
|  | Exclusion criteria | Unable to provide informed consent, no means of obtaining adequate biological samples, diagnosed with hepatitis C, and under specific treatment |
| **HPV** | Inclusion criteria | Workers undergoing OHS, HPV vaccination eligibility 18-45 years of age. |
|  | Exclusion criteria | Unable to provide informed consent, severe allergic reaction (e.g., anaphylaxis) after a previous dose or to a vaccine, component, Pregnancy, Moderate or severe acute illness with or without fever |
