# Supplementary material for: Cancer Prevention at Work (CPW) project: Rationale, framework and research protocol
Source: PLoS One. 2025 Nov 3;20(11):e0335752. doi: 10.1371/journal.pone.0335752 (PMC12582473; doi:10.1371/journal.pone.0335752)
Supplement: S3 Table — (DOCX) [file pone.0335752.s003.docx]

**Supplementary Table 3. Details of baseline and follow-up questionnaires for HP, HCV, and HPV interventions**

| **Questionnaire type** | **Section** | **Detailed variables** |
| --- | --- | --- |
| **Baseline questionnaires** | **List of common variables between the three interventions (HP-HCV-HPV)** | |
|  | **Demographic information** | Sex, age, height, weight, ethnicity, place of residence, blood group |
|  | **Socioeconomic status** | Education, marital status, number of family members, and foreign language |
|  | **Dietary items** | Consumption of: Meat intake (red, white, processed meat, fish), fruit and vegetables, dairy products, snacks (cookies, biscuits, etc), soft drinks.  Food habit questions: salt intake, spicy food, food preparation method, coffee intake |
|  | **Occupational history** | Job title, duration of employment |
|  | **Medical history** | Diabetes, hypertension, Hepatitis B/C/D, HIV, sexually transmitted infection, Renal failure, Haematological disorders, Organ/tissue/ cell transplant, cancer |
|  | **Family history** | Relationship type, age at diagnosis, cancer type |
|  | **Tobacco and alcohol use** | Type of smoking, second-hand smoking, age at start, age at smoking cessation, frequency of alcohol drinking, type of alcohol drink, amount of alcohol intake |
|  | **Self-perceived health** | Healthy diet, individual health status, saving / financial funds, friendship status |
|  | **List of specific variables for HP intervention (WP2)** | |
|  | **Occupational history** | +Night shift, hygiene at work, a place where food is kept/served |
|  | **Dietary items** | **+Diet, Probiotics, Salt, Coffee, Alcohol, Tobacco,** drinking and household water source, energy drinks intake, the place for food intake (out of home, take away, restaurants, etc)**,** washing hands before meal intake |
|  | **Medical history** | +Mental disease, Mononucleosis, Tuberculosis, Pernicious anaemia, Fungal infections, Neurological, Immunodeficiency disease, Inflammatory bowel disease, Thyroid disease, Allergy, Ulcer, Gastro-oesophageal reflux, Gastritis, Gastric Polyps, MALT Lymphoma, Helicobacter Pylori infection, Hepato-intestinal, gastric endoscopic interventions, undergone stomach surgery, Pharmacological history |
|  | **Emotional stress** | Related to the workplace situation, |
|  | **Hp knowledge** | General knowledge of gastric cancer risk factors, risks of HP infection, history of HP screening, testing, or therapy |
|  | **Symptoms-pharma** | Gastric symptoms history (Epigastric pain/discomfort, Retrosternal burning, Nausea, etc.), medicine use history ( Antacids, NSAIDs, Antibiotics, etc.) |
|  | **List of specific variables for HCV intervention (WP3)** | |
|  | **Occupational history** | +Exposure to biological hazards, injury with biological contamination risk |
|  | **Risk behaviours and others** | Drug (Marijuana, Tranquillizers or sedatives, Amphetamines, etc.), sexually transmitted diseases (STD) conditions, and type of them if yes (HIV, syphilis, etc.), sexual activity status/ partners, been in prison expertise, |
|  | **Risk factors and tests** | Blood transfusion, treatment with blood  Derivatives/components, dialysis treatment, endoscopic procedures, tattoos or piercings, blood donor, vaccinated against Hepatitis B, vaccinated against Human papillomavirus (HPV), |
|  | **HCV knowledge** | Level of awareness of risk factors for liver cancer, HCV infection, |
|  | **List of specific variables for HPV intervention (WP4)** | |
|  | **Occupational history** | +Exposure to biological hazards, injury with biological contamination risk |
|  | Medical history | + Pregnancy at the interview time |
|  | **Risk behaviours and others** | Drug (Marijuana, Tranquillizers or sedatives, Amphetamines, etc.), sexually transmitted diseases (STD) conditions and type of them if yes (HIV, syphilis, etc.), sexual activity status/ partners, been in prison expertise, |
|  | **HPV knowledge** | General knowledge of HPV, Level of awareness of cancer outcomes related to HPV (cervix uteri, vagina, Valva, penile, anal and rectal, head and neck), vaccination programs, screening and testes for HPV infection, |
|  | **Vaccine knowledge** | General knowledge of HPV vaccination, history of vaccination or family member vaccination status, willingness to vaccination, or reasons for refusal. |
| **Follow up questionnaire** | **List of common variables between three interventions (HP-HCV-HPV)** | |
|  | Participants' age, follow-up date, date of positive test, and reasons for refusal, if they do not agree to follow the study. | |
|  | **List of specific variables for HP intervention (WP2)** | |
|  | Contact details for health system referral after a positive test for evaluation, Training of occupational and primary care health professionals, , Follow-up to positive workers after six months. Symptoms related to the therapy, Benefits of ending the treatment, Patient's willingness to follow up and continue treatment, Effects of the therapy on gastrointestinal health, Family members' awareness of the situation and their willingness to take an HP test, Any changes in eating habits. | |
|  | **List of specific variables for HCV intervention (WP3)** | |
|  | Status of medical recommendations following HCV infection or liver disease diagnoses, including reasons for acceptance or refusal, current treatment status for HCV infection, overall view on the study and protocol | |
|  | **List of specific variables for HPV intervention (WP4)** | |
|  | Status of medical recommendations following regarding HPV vaccination, detail of vaccination (dose, date, place, costs, side effects), family member vaccination status, overall view on the study and protocol. | |
